# Genetic Variants in pre-miR-146a, pre-miR-499, pre-miR-125a, pre-miR-605, and pri-miR-182 Are Associated with Breast Cancer Susceptibility in a South American Population

**DOI:** 10.3390/genes9090427

**Published:** 2018-08-22

**Authors:** Sebastián Morales, Tomas De Mayo, Felipe Andrés Gulppi, Patricio Gonzalez-Hormazabal, Valentina Carrasco, José Miguel Reyes, Fernando Gómez, Enrique Waugh, Lilian Jara

**Affiliations:** 1 Human Genetics Program, Institute of Biomedical Sciences (ICBM), School of Medicine, University of Chile, Santiago 8380453, Chile; seba.morales.p@gmail.com (S.M.); felipe.gulppi@gmail.com (F.A.G.); pgonzalez@med.uchile.cl (P.G.-H.); valentina.carrasco@gmail.com (V.C.); 2 Center for Genetics and Genomics, School of Medicine, Clínica Alemana Universidad del Desarrollo, Santiago 7610658, Chile; tdemayog@udd.cl; 3 Clínica Las Condes, Santiago 7591047, Chile; jmreyes@clc.cl; 4 Clínica Santa María, Santiago 7520378, Chile; fgomez@csm.cl (F.G.); ewaugh@csm.cl (E.W.)

**Keywords:** familial breast cancer, polymorphisms, microRNA, South American population

## Abstract

Breast cancer (BC) is one of the most frequent tumors affecting women worldwide. microRNAs (miRNAs) single-nucleotide polymorphisms (SNPs) likely contribute to BC susceptibility. We evaluated the association of five SNPs with BC risk in non-carriers of the *BRCA1/2*-mutation from a South American population. The SNPs were genotyped in 440 Chilean *BRCA1/2*-negative BC cases and 1048 controls. Our data do not support an association between rs2910164:G>C or rs3746444:A>G and BC risk. The rs12975333:G>T is monomorphic in the Chilean population. The pre-miR-605 rs2043556-C allele was associated with a decreased risk of BC, both in patients with a strong family history of BC and in early-onset non-familial BC (Odds ratio (OR) = 0.5 [95% confidence interval (CI) 0.4–0.9] *p* = 0.006 and OR = 0.6 [95% CI 0.5–0.9] *p* = 0.02, respectively). The rs4541843-T allele is associated with increased risk of familial BC. This is the first association study on rs4541843 and BC risk. Previously, we showed that the *TOX3*-rs3803662:C>T was significantly associated with increased risk of familial BC. Given that *TOX3* mRNA is a target of miR-182, and that both the *TOX3* rs3803662-T and pri-miR-182 rs4541843-T alleles are associated with increased BC risk, we evaluated their combined effect. Risk of familial BC increased in a dose-dependent manner with the number of risk alleles (*p*-trend = 0.0005), indicating an additive effect.

## 1. Introduction

Breast cancer (BC) is one of the most frequent cancers affecting women worldwide. One of every eight women will develop BC in their lifetime [1]. In Chile, BC has the highest mortality rate among cancers (15.69/100,000 women), and its incidence is rising in all of the age groups monitored [2]. The mutations in the *BRCA1/2* genes are responsible for an average of 16–20% of the risk for hereditary BC [3,4,5]. Moreover, it has been proposed that BC susceptibility alleles can be classified into three categories of penetrance (high, moderate, or low) that reflect the probability of developing the disease [6]. Our group has studied the contribution of moderate- and low-penetrance genes (*PALB2* [7], *BARD1* [8], *ATM* [9], *CHECK2* [10], *FGFR2* [11], *TOX3* [12], *MAP3K1* [11], and *8q24* [12]) to genetic susceptibility to hereditary BC in the Chilean population. As genetic factors play an important role in BC etiology [13], identifying the genetic alterations involved in breast carcinogenesis is a major priority in the field. 

In recent years, evidence has emerged to support a role for microRNAs (miRNAs) in BC development and progression [14,15]. miRNAs are single-stranded RNAs of about 22 nucleotides in length. These molecules can regulate gene expression by degrading or blocking the translation of their specific target mRNAs, mainly by binding to their 3′-untranslated region (UTR) [16,17]. Approximately 30% of all of the human genes are regulated by miRNAs [18,19]. Growing evidence has established that miRNA misexpression and mutations are correlated with various human cancers, including BC [20,21,22]. Single-nucleotide polymorphisms (SNPs) are the most common type of variation in the human genome. The SNPs present in miRNAs can alter the expression, lead to the maturation of aberrant miRNA, and affect the target binding affinity and specificity. Therefore, these variants may contribute to some forms of familial cancer [23]. Many epidemiological studies have examined the association between miRNA SNPs and cancer susceptibility [22]. In BC, several case-control studies and meta-analyses have evaluated the association between the miRNA gene polymorphisms and disease risk in European [24,25,26,27,28,29], Asian [30,31], Arab [32], Jewish [33], and Iranian populations [34]. With the exception of two studies, one in a Brazilian [35] and the other in Chilean population published by our group [36], the contribution of miRNA gene variants to BC risk among South American women remains unexplored. We have previously studied the contribution of the SNPs rs895819 (pre-miR-27a), rs11614913 (pre-miR-196a2), rs6505162 (pre-miR-423), rs4919510 (miR-608), and rs2682818 (pre-miR-618) to BC susceptibility in the Chilean population, as these were the most-studied SNPs in the BC cases from several populations. Moreover, the genetic variability is ethnicity-specific and appears to influence not only the risk but also the type of BC that develops in an individual. In this study, we selected SNPs in three miRNAs (rs3746444 in pre-miR-499, rs12975333 in pre-miR-125a, and rs2043556 in miR-605), based on the evidence in the literature for a relationship with BC risk [23,24,31,34,37,38,39]. Another four miRNAs (pre-miR-16, pre-miR-182, pre-miR-192, and pre-miR-146a) were also selected for analysis, as these miRNAs are known to target *BRCA1/2* genes [40,41,42,43]. Therefore, in this study, we sequenced the complete coding regions and boundaries of pre-miR-16, pre-miR-182, pre-miR-192, and pre-miR-146a in a population of Chilean BC patients who were negative for *BRCA1* and *BRCA2* mutations, with the aim of identifying new variants. The patients had either a strong family history of BC or of early-onset BC. The SNPs were identified in only two of the miRNAs sequenced, pre-miR-146a (rs2910164) and pri-miR-182 (rs4541843). A case-control designed was used to assess the association between BC risk and SNPs, rs3746444 (pre-miR-499), rs12975333 (pre-miR-125a), rs2043556 (miR-605), rs2910164 (pre-miR-146a), and rs4541843 (pri-miR-182). 

The human miR-146a gene at locus 5q34 has been linked with *BRCA1/BRCA2* activity. The SNP rs2910164:G>C, located in the middle of the miRNA stem hairpin, leads to a change from a G:U pair to a C:U mismatch in the stem structure of the precursor molecule, altering the expression of mature miR-146a [44]. This SNP has been associated with the risk of various cancers [45,46], and with cancer-specific and ethnicity-dependent effects [23,37].

The variant rs3746444 in the mature miR-499-3p produces a change from an A:U pair to a G:U mismatch in the stem structure of the precursor molecule, leading to an altered processing and expression of the mature transcript [44], and potentially affecting the binding of the target mRNAs to the mature miRNA-3p [44]. miR-499 can target regulation of *FOXO4*, *PDCD4*, *Sox6*, and *Rod1* expression [47,48,49], all of which play important roles in the etiology of various cancers [48]. Many studies have explored the association between the rs3746444:A>G SNP and the susceptibility to BC [44], lung cancer [50], gallbladder cancer [51], squamous cell carcinomas of the head and neck [52], liver cancer [53], and colorectal cancer [54]. Studies on the association between this SNP and BC susceptibility have also shown that rs3746444 has different effects on different populations.

miR-125a is located on chromosome 19q13.41 in the human genome. The identified targets of miR-125a include *Lin-28*, *Lin-41*, *ERBB2*, and *ERBB3* mRNAs, all of which are involved in BC tumorigenesis [55,56,57]. One miRNA profiling study found that mir-125a was downregulated in BC [58]. Other data suggest that miR-125 may play an important role in BC pathogenesis [59]. The rs12975333:G>T is located at the eighth nucleotide (+8) within the mature miR-125a. An in vivo analysis demonstrated that this SNP significantly blocks the processing of pri-miRNA to pre-miRNA, and reduces the miRNA-mediated translational suppression [59]. This SNP was also strongly associated with BC tumorigenesis in a Belgian population from Antwerp [38], suggesting that miR-125a likely functions as a tumor suppressor gene in human cancer [38]. However, in other populations, this SNP is monomorphic for the wild-type allele [39].

Several studies have investigated the association between miR-605 rs2043556:T>C and cancer risk. A meta-analysis by Hu et al. [60], concluded that there was a significant association between the rs2043556 C allele and an overall risk of human cancer. To date, however, only two association studies have been performed to assess the contribution of miR-605 rs2043556 to BC risk [23,31].

Finally, the rs4541843:C>T located in the boundaries of pre-miR-182 (pri-miR-182 region), and is described in the Ensembl genome browser 90 database. While miR-182 is known to be involved in breast carcinogenesis, there are no association studies in the literature regarding the contribution of rs4541843 to BC susceptibility.

This study was designed to evaluate the association of these SNPs rs2910164 in pre-miR-146a, rs4541843 in pri-miR-182, rs3746444 in pre-miR-499, rs12975333 in pre-miR-125a, and rs2043556 in miR-605 with familial BC and early-onset non-familial BC in non-carriers of *BRCA1/2* mutations from a South American population.

## 2. Materials and Methods 

### 2.1. Families

A total of 440 BC patients belonging to 440 high-risk, *BRCA1/2*-negative, Chilean families were selected from the files of the Servicio de Salud del Area Metropolitana de Santiago, Corporación Nacional del Cáncer (CONAC), and other private health services in the Metropolitan Region of Santiago. All of the index cases were tested for *BRCA1* and *BRCA2* mutations, as previously described [61]. Pedigrees were constructed from the index case with the highest probability of carrying a deleterious mutation. None of the families studied met the criteria for other known BC-related syndromes, such as Li-Fraumeni, ataxia-telangiectasia, or Cowden disease. 

Table 1 shows the specific characteristics of the families selected according to the inclusion criteria. All of the study families had self-reported Chilean ancestry dating from several generations, confirmed by extensive interviews with several family members from different generations. A total of 16% of the families (70/440) had bilateral BC cases, 9.0% (40/440) had both BC and ovarian cancer (OC) cases, and 1.1% (5/440) had male BC cases. The mean age at diagnosis for the BC cases was 42.1 years, and 75.2% were diagnosed at <50 years.

The study was approved by the Institutional Review Board of the University of Chile, School of Medicine (Project code Number 1150117, 1 March 2015). Informed consent was obtained from all of the participants.

### 2.2. Control Population

The sample of healthy Chilean controls (*n* = 1048) was recruited from CONAC files. Only individuals whose ancestors were Chilean for at least the three previous generations were included in the study. The DNA samples were taken from unrelated individuals with no personal or family history of cancer, confirmed by interview performed by a geneticist of our research group. All of the participants provided informed consent for anonymous testing. The DNA samples were obtained in compliance with all of the relevant ethical and legal norms. The control sample was matched to cases for age and socioeconomic strata. Over 90% of the cases and controls lived in the city of Santiago.

### 2.3. Mutation Analysis

Genomic DNA was extracted from peripheral blood lymphocytes of 440 cases from the selected high-risk families and 1048 controls. The samples were obtained according to the method described by Chomczynski and Sacchi [62].

A complete sequencing study was performed for pre-miR-16 (Chr13:50,048,973–50,049,061), pre-miR-182 (Chr7:129,770,383–129,770,492), pre-miR-192 (Chr11:64,891,137–64,891,246), and pre-miR-146a (Chr5:160,485,352–160,485,450), as well as the boundaries (100 pb to each side) sequence in 99 of the 440 cases. For this analysis, the families were subdivided into those with (a) three or more members with BC and/or OC (43.4%) and (b) index cases with early-onset BC (≤35 years) (56.6%). The entire coding sequence and the boundaries of the pre-miRNA(s) sequences were amplified by polymerase chain reaction (PCR). The primers were designed with Primer3 version 0.4.0 [63]. The sequencing was performed using an ABI 3730xl automated fluorescence-based sequencer and BigDye Terminator v3.1 kit (Applied Biosystems, Foster City, CA, USA).

The SNPs, rs3746444 (A>G), rs2910164 (G>C), rs12975333 (A>C), and rs2043556 (T>C), were genotyped using commercially-available TaqMan Genotyping Assays (Thermo Fisher Scientific, Fair Lawn, NJ, USA) (assay ID C__2142612_30, C__15946974_10, C__31444793_10, and C__11737438_10, respectively). A custom TaqMan assay was designed to genotype the SNP rs4541843 (context sequence 5′-ACAGCCAGCGAGGGAAGGGC[C/T]GGCCAATGCTGGACCTGCTGTT-3′). The reaction was performed in a 10 μL final volume containing 5 ng of genomic DNA, 1X TaqMan Genotyping Master Mix, and 20X TaqMan SNP Genotyping Assay. The PCR was carried out in a StepOnePlus Real-Time PCR System (Applied Biosystems, Foster City, CA, USA). The thermal cycles were initiated for 10 min at 95 °C, followed by 40 cycles, each at 92 °C for 15 s and 60 °C for 1 min. Each genotyping run contained control DNA confirmed by sequencing. The alleles were assigned using StepOne software, v2.2 (Applied Biosystems, Foster City, CA, USA). As a quality control, we repeated the genotyping on ~10% of the samples, and all genotype scoring was performed and checked separately by two reviewers unaware of case-control status.

### 2.4. Statistical Analysis

The Hardy–Weinberg equilibrium assumption was assessed in the control sample using a goodness-of-fit chi-square test (HW Chisq function included in the ‘HardyWeinberg’.package v1.4.1 for R, Foundation for Statistical Computing, Vienna, Austria, URL: https://www.r-project.org/). The Fisher’s exact test was used to test the association between the genotypes/alleles and the case/control status. The odds ratios (OR) with 95% confidence intervals (CI) were calculated to estimate the strength of the associations (odds ratio and Fisher’s exact test functions were performed using GraphPad Prism v 6.0 for Windows 10, GraphPad Software, La Jolla California, USA, URL: www.graphpad.com) A two-tailed *p*-value <0.05 was used as the criterion of significance. The Cochran–Armitage trend test was performed to test the additive genetic effect model (CATT function included in the ‘Rassoc’ package v 1.03 for R, Foundation for Statistical Computing, Vienna, Austria, URL: https://www.r-project.org/). A chi-square test for the trend was performed to examine the additive combined effects of the risk alleles (‘ptrend’ was performed in Stata/MP v 13.0 for Windows 10, Unix-StataCorp, College Station, TX, USA; using ‘ptrend’ package). 

## 3. Results

### 3.1. Association Study between rs2910164, rs4541843, rs3746444, rs12975333, and rs2043556 with Familial Breast Cancer and Early-Onset Non-Familial Breast Cancer in Non-Carriers of BRCA1/2 Mutations

We analyzed the complete coding sequence and boundaries of pre-miR-16, pre-miR-182, pre-miR-192, and pre-miR-146a in 99 probands who had a strong family history of BC, but were negative for *BRCA1* and *BRCA2* point mutations, with the aim of identifying new miRNA sequence variations in a Chilean population. No variants were detected in the pre-miR-16 and pre-miR-192 sequences. We identified two variants, rs2910164 (pre-miR-146a) and rs4541843 (pri-miR-182). These two variants and three SNPs from the literature, rs3746444 (pre-miR-499), rs12975333 (pre-miR-125a), and rs2043556 (miR-605), were then analyzed in 440 *BRCA1/2*-negative cases and 1048 controls. For the case-control analysis, the whole BC sample was subdivided into two subgroups, individuals from families with two or more members with BC and/or OC (*n* = 269) (subgroup A), and individuals with non-familial early-onset BC (≤50 years) (*n* = 171) (subgroup B). Subgroup A excludes the subgroup B cases. The genotype distributions and allele frequencies of pre-miR-146a rs2910164:G>C, pri-miR-182 rs4541843:C>T, pre-miR-499 rs3746444:A>G, pre-miR-125a rs12975333:G>T, and miR-605 rs2043556:T>C are shown in Table 2 for the whole patient group and subgroups vs. the controls. The observed genotype frequencies for three of the five polymorphisms were in Hardy-Weinberg equilibrium in the controls (*p* = 0.86 for rs2910164:G>C, *p* = 0.83 for rs3746444:A>G, and *p* = 0.90 for rs12975333), while the *p*-values for the SNPs rs4541843:C>T and rs2043556:T>C were 0.03 and <10^−4^, respectively.

In the single-locus analysis, the genotypes and alleles distributions for rs3746444:A>G, did not differ significantly between cases and controls, in either the whole-group or subgroup analysis (*p* > 0.05). The genotype frequencies of rs2910164:G>C in the 99 *BRCA1/2*-negative probands from high-risk families were 52.7% G/G, 39.4% G/C and 7.9% C/C. For the case-control analysis of rs2910164:G>C, the genotypes and alleles distributions did not differ significantly between cases and controls, in either the whole-group or subgroup analysis (*p* > 0.05). For rs12975333:G>T, the frequency of the wild-type allele (G) was 0.996 in controls and 0.995 in cases, and the frequency of allele T was 0.004 in controls and 0.005 in cases. Therefore, the T allele was extremely rare and practically monomorphic in this Chilean population. 

The rs2043556:T>C is located in pre-miR-605. The minor allele frequency (MAF) (allele C) was significantly lower in the whole sample (0.32) and in the subgroup A (0.31) vs. controls (0.37) (OR = 0.8 [95% CI 0.6–0.9] *p* = 0.01 and OR = 0.7 [95% CI 0.6–0.9] *p =* 0.01, respectively). This result indicates that the C allele is associated with a protective effect against BC risk. We also observed a protective effect for the C-allele carriers (T/C + C/C) in the whole sample (OR = 0.6 [95% CI 0.4–0.7], *p* < 10^−4^), in subgroup A (OR = 0.6 [95% CI 0.4–0.8] *p* < 10^−4^), and in single cases diagnosed at ≤50 years of age (subgroup B) (OR = 0.6 [95% CI 0.5–0.9] *p* = 0.02). We also assessed for a protective effect of rs2043556 in according to number of BC cases in the family (Table 3). No protective effect was found for rs2043556 in the families with two BC/OC cases. However, the BC risk was significantly decreased in the C-allele carriers with three or more family members with BC/OC (T/C + C/C) (OR = 0.5 [95% CI 0.4–0.9] *p* = 0.006). This result indicates that the C-allele was associated with a protective effect in the families with a strong history of BC. 

The rs4541843:C>T corresponds to a SNP detected by sequencing in pri-miR-182. This SNP is located at the position 138 downstream of 5′ pre-miR-182. The genotype frequencies in the 99 *BRCA1/2*-negative probands from high-risk families were 34.6% C/C, 42.7% C/T, and 22.7% T/T. In the case-control analysis, no significant differences were observed for genotype or allele distribution, for whole group or subgroup B vs. controls (*p* > 0.05). However, in the familial BC cases (subgroup A), the MAF (allele T) was higher in cases than controls (0.46 and 0.41, respectively, *p* = 0.01). Furthermore, in subgroup A, the homozygous T/T- and T-allele carriers (C/T + T/T) had a significantly increased BC risk (OR = 1.5 [95% CI 1.0–2.2] *p* = 0.03 and OR = 1.2 [95% CI 1.0–1.5] *p* = 0.01, respectively) (Table 2), indicating that the T allele is associated with an increased BC risk. When we analyzed the effect of the T allele by the number of BC cases per family, no association between the rs4541843 and BC risk was found. No associations were observed between this SNP and the early-onset BC (diagnosis ≤50 years of age). It is important to note that is the first association study on this SNP and the risk of a human disease. 

### 3.2. Combined Effect between TOX3 rs3803662-T and pri-miR-182 rs4541843-T Alleles with Breast Cancer Risk

In a previous publication, our group showed that *TOX3* rs3803662:C>T was significantly associated with an increased BC risk in familial BC [12]. As *TOX3* rs3803662-T and pri-miR-182 rs4541843-T were associated with an increased BC risk, we considered these two variants to be risk alleles. Moreover, *TOX3* mRNA is a target of miR-182 [64]. Therefore, we evaluated the combined effect of these variants. For the analysis, the subjects were divided into five groups based on number of risk alleles (subjects with 0 [group 1], one [group 2], two [group 3], three [group 4], or four [group 5] risk alleles). As shown in Table 4, the distribution of the combined genotypes in the whole patient group and in subgroup A significantly differed from the controls (*p* = 0.005 and 0.0001, respectively), and the BC risk increased in a dose-dependent manner in the whole sample, and in subgroup A, with the number of risk alleles (*p*-trend = 0.0005 and <10^−4^, respectively). No additive effect was observed for early-onset BC (diagnosis ≤50 years of age). We also analyzed this additive effect within cases with a family history of BC, according to the number of BC cases per family (Table 5). An additive effect was observed in the families with two BC/OC cases and the families with the strongest history of BC (*p*-trend = 0.0001 and 0.001, respectively). These results indicate an additive effect of *TOX3* rs3803662-T and pri-miR-182 rs4541843-T on BC risk.

## 4. Discussion

Currently, there is consensus that the *BRCA1* and *BRCA2* mutations are responsible for an average of only 16% of the risk for familial breast and ovarian cancers [3,5]. Consequently, there is an intensive search for additional targets. 

The miRNAs are a class of endogenous, non-coding, single-stranded RNAs involved in many molecular pathways and biological processes, including the development, apoptosis, differentiation, proliferation, and immune response [65]. Many miRNAs have been implicated in various human diseases, and it has been shown that miRNAs are aberrantly expressed or mutated in many cancers. SNPs are the most common form of variation present in the human genome. The SNPs in the miRNA regions can alter the genetic expression, processing, and maturation, as well as the target binding affinity and specificity [23,66]. Therefore, many epidemiological studies have examined the associations between the miRNA SNPs and BC susceptibility [22]. It is important to note that genetic variability is ethnicity-specific. To date, most miRNA SNP studies have been performed in cases from European, Asian, Arab, or Jewish populations, mainly in sporadic BC. In contrast, the publications regarding the role of the miRNA variations in BC susceptibility among Latin-American populations are very scarce. In the present study, we evaluated the impact of the miRNA SNPs on familial and early-onset BC in Chilean families negative for *BRCA1/2* point mutations. To this end, we performed a case-control study to examine the association between the BC risk and rs2910164 in pre-miR-146a, rs4541843 in pri-miR-182, rs3746444 in pre-miR-499, rs12975333 in pre-miR-125a, and rs2043556 in pre-miR-605. 

Our data do not support an association between rs2910164:G>C or rs3746444:A>G and BC risk. The SNP rs2910164:G>C resides within miR-146a, a microRNA that binds to the 3′ untranslated region of the *BRCA1* transcript [43] and negatively regulates expression of this gene [27]. There have been a number of studies on rs2910164 in populations of various ethnicities, but the results are controversial [24,25,27,43,67,68,69]. Shen et al. (2008) [43] reported in US population that *BRCA1/*2-negative BC/OC cases with G/C-C/C genotypes were significantly younger at diagnosis than those with the G/G genotype; nevertheless, the ethnicity of the US population used in this study was not specified. This association was not confirmed in a study by Catucci et al. (2010) [24], who reported no association between rs2910164 with BC risk or the age of onset in *BRCA1/2*-negative BC patients from Germany and Italy. On the other hand, Pastrello et al. (2010) [25] reported that the SNP miR-146a rs2910164 had a potential impact on the age of onset in an Italian population of *BRCA1/2*-negative familial BC/OC patients. In a North Indian population, Bansal et al. (2014) [68] demonstrated that the miR-146a G/G (rs2910164) polymorphism was associated with reduced genetic susceptibility to BC. However, a multivariate analysis showed that this SNP was associated with an increased BC risk in postmenopausal females. In a Chinese population, the rs2910164 G/G and C/G-G/G genotypes were associated with an increased BC risk in the postmenopausal BC cases with no familial history of BC [69]. In an Iranian population, the rs2910164 polymorphism was not significantly associated with the occurrence of BC. Several meta-analyses that included the rs2910164:G>C polymorphism were published between 2011 and 2017. Of these, two meta-analyses reported that rs2910164 contributed to BC susceptibility in a Caucasian population [29,70]. The other five meta-analyses showed that rs2910164 was not associated with BC susceptibility in Caucasians or Asians [23,37,44,71,72]. The majority of authors suggest that the oncogenic mechanisms are markedly influenced by specific genetic backgrounds across populations. The contemporary Chilean population stems from the admixture of Amerindian people with the Spanish settlers in the sixteenth and seventeenth centuries. Later (nineteenth-century) migrations of Germans, Italians, Arabs, and Croatians had only a minor impact on the general population (accounting for no more than 4% of the total population), with effects restricted to the specific locations of the country where these immigrants settled [73]. The relationships among ethnicity, Amerindian admixture, genetic markers, and socioeconomic strata in Chile have been studied extensively [74,75]. The SNP rs3746444, located at the 3p mature miRNA-499, was also found to have no association with BC risk in our study. This polymorphism involves an A>G nucleotide substitution, leading to altered processing and the expression of the mature transcript [76]. The analyses of rs3746444 polymorphisms in BC patients have shown mixed results. Several studies have reported that rs3746444 was associated with an increased BC risk in Iranian [34,67,77] and Chinese populations [69,78]. In contrast, other studies found no association between rs3746444 and BC risk in Caucasian (German and Italian) [24] or North Indian populations [68]. In addition, all five meta-analyses published to date have found that rs3746444 is associated with an increased BC risk in Asians, but not Caucasians [23,44,72,76,79]. Given the European and Native American ancestry estimates for Chileans as 52% and 44% on average, respectively, these results could explain the lack of association of rs3746444 with BC risk in this group [80]. 

The variant rs12975333:G>T is located in the seed region of the mature miRNA-125a. The T allele reportedly blocks the processing of pri-miRNA to the pre-miRNA precursor. This variation is extremely rare, having been detected in only one of 1200 individuals from diverse ethnic backgrounds in a study by the Centre d’Etude du Polymorphisme Humain [38]. Li et al. (2009) [38] showed that rs12975333-T was strongly associated with BC risk in a Belgian population from Antwerp. These authors found that while 8.3% of the BC cases were T-allele carriers, this risk allele was not present in any of the 192 controls from the general population in the Antwerp area or the 587 Caucasian controls collected in the United States [38]. This SNP was also monomorphic for the G allele in a sample of 340 healthy individuals from Catalonia (in northeast Spain) [81]. Peterlongo et al. (2011) [39] showed that rs12975333 was monomorphic in 4114 controls from Germany, Italy, Australia, and Spain (Madrid). Moreover, the authors suggested that very few BC cases, if any, were attributable to rs12975333 in the populations studied. Our results also indicate that rs12975333 is extremely rare, if not absent, in the BC cases and controls from a Chilean population. Therefore, we were unable to estimate the association between rs12975333 and BC risk.

Several studies have investigated the association between miR-605 rs2043556:T>C and cancer risk [82]. Specifically, the miR-605 C allele was associated with an increased risk of bladder cancer in a Caucasian population [60], and in gastrointestinal cancer, the C allele was significantly less common in the controls vs. cancer patients in an Asian population [31]. However, rs2043556-C significantly decreased the oral squamous cell carcinoma risk in a Chinese population [82]. Only one article and one meta-analysis have evaluated the association between rs2043556 and BC risk. In a Chinese population, no significant association with BC risk was observed [31], while the meta-analysis by Chen et al. (2014) [23], reported that the miR-605 rs2043556 C/C genotype may increase the BC susceptibility in an Asian population. Because all of the participants in this meta-analysis were Asian, additional case-control studies, especially in non-Asian populations, are necessary to validate the finding. Our results showed that the rs2043556 C allele was associated with a protective effect in the *BRCA1/2*-negative Chilean women with a strong family history of BC or non-familial, early-onset BC. These results are contradictory to the results obtained in Asians. Therefore, given that the Chilean population is ~52% Caucasian and ~44% Native Amercican, studies in other population are needed. One important issue to consider is that the genotype distribution of rs2043556 is in a Hardy–Weinberg disequilibrium, which could distort the results. The possibility that different selective factors may directly or indirectly alter the association between rs2043556 and BC risk cannot be discarded.

miR-182 is one of three miRNAs in the miR-183/182/96 cluster, located in a 5-Kb region of human chromosome 7q32.2 [83]. Several studies have confirmed that the members of the miR-182 cluster are abnormally expressed in some cancers and other human diseases [84]. This cluster, and specifically miR-182, is highly expressed in many BC subtypes. This molecule functions as an onco-miRNA, promoting the proliferation and migration of BC cells [85]. With respect to miR-182, Moskwa et al. (2011) [41] reported that the Argonaute/miR-182 complex associates selectively with the BRCA1 transcript and that miR-182 downregulates the *BRCA1* expression. Therefore, miR-182 overexpression reduces the BRCA1 protein levels and impairs the homologous recombination-mediated repair. In addition, consistent with a BRCA1-deficiency phenotype, miR-182-overexpressing breast tumor cells are hypersensitive to the inhibitors of poly (ADP-ribose) polymerase I (PARP1) [41]. Krishman et al. (2013) [86] showed that miR-182 is overexpressed in several molecular subtypes of BC. This author then experimentally validated that miR-182 mediates the disruption of homologous recombination (HR), as a consequence of its ability to target multiple components of that pathway [86]. Other targets of miR-182 include the transcription factor *TOX3* mRNA; Nibrin *NBN* mRNA, which is a gene member of the MRE11/RAD50 double-strand break repair complex; and LIM and SH3 domain protein 1 (*LASP1)* mRNA. Therefore, miR-182 may participate in the regulation of DNA double-strand break repair and estrogen receptor-mediated gene expression, by regulating the *NBN* and *TOX3* expression. Moreover, miR-182 may also influence the nodal positivity and tumor size of breast carcinomas by regulating *LASP1* expression [64]. The SNP rs4541843:C>T is located in pre-miR-182. This SNP is described in the Ensembl genome browser 90 database; nevertheless, there are no studies in the literature assessing the association of rs4541843:C>T with any human disease. Here, we performed a case-control study on rs4541843:C>T, showing that the T allele is associated with an increased BC risk. In addition, homozygous T/T- and T-allele carriers (C/T + T/T) had a significantly increased BC risk among *BRCA1/2*-negative familial BC cases. Given the known roles of miR-182, it could be hypothesized that rs4541843-T induces miR-182 overexpression, reducing the BRCA1 protein levels by altering the pathways involved in maintaining the genomic stability. Thus, the association of rs4541843-T with an increased BC risk in the *BRCA1/2*-negative BC patients could be a consequence of reduced BRCA1 protein levels. Studies in other populations are needed to confirm this assertion, as this is the first association study on rs4541843:C>T and BC risk.

In a previous publication, our group described an association between the *TOX3* rs3803662:C>T and risk of familial BC. In this study, we showed that rs4541843-T, located in the boundaries of pre-miR-182 (pri-miR-182 region), is also associated with an increased BC risk. Considering that the transcription factor *TOX3* mRNA is a target of miR-182 [64], we evaluated the combined effects of the *TOX3* rs3803662-T and pri-miR-182 rs4541843-T, and constructed a genetic score based on the number of risk alleles. A dose-response association was observed for familial BC. The presence of four risk alleles was associated with a 3.2-fold increased risk of familial BC compared with the zero risk alleles. miRNA-182 is an onco-miRNA in BC [85,87] that regulates the expression of *BRCA1*, *NBN*, *LASP1*, and *TOX3*, and other genes [64]. miR-182 is upregulated in BC, increasing the proliferation, migration, and tumorigenesis of BC cells [87,88]. *TOX3* also encodes a protein that plays a pivotal role in calcium-dependent transcription, as a transcription factor [89]. This gene is located on chromosome 16q12. The loss of heterozygosity (LOH) and the translocations involving the 16q region are commonly observed in BC [90]. Moreover, *TOX3* is expressed in mammary ER+ epithelial cells, and regulates the expression of estrogen receptor-mediated genes [91]. Associations between several *TOX3* or nearly *TOX3* SNPs and BC susceptibility have been established in European, Asian, African American, and South American populations [12,92,93,94,95,96]. Among these, rs3803662:C>T is the most strongly correlated with disease. Each copy of rs3803662-T is associated with a 20% increase in BC risk [93]. Riaz et al. (2012) [97], suggested that *TOX3* might act as a tumor suppressor gene and that the risk allele rs3803662-T is significantly associated with a reduced *TOX3* expression. Furthermore, *TOX3* mRNA is a target of miR-182, and miR-182 overexpression reduces the *TOX3* endogenous transcript level [64]. Consequently, the presence of the risk alleles from both of the SNPs could produce a more marked decrease in the *TOX3* expression. This effect increases with the number of risk alleles, producing a dose-dependent increase in the BC risk. Functional studies are needed to elucidate the biological consequences of rs4541843:C>T in breast and other cancers. The potential utility of miR-182 in BC therapy should also be considered. Although our study provides evidence for an association of rs2043556 and rs4541843 with BC risk, certain limitations must be considered. Firstly, the genotype distribution of rs2043556 did not conform to the Hardy–Weinberg expectations, which may distort the results. Secondly, the sample size of the whole group in the present study is sufficient to yield 80% power; nevertheless, the sample size limits the subgroup analyses. Therefore, these results should be replicated using subgroups with larger sample sizes.

## Figures and Tables

**Table 1:** Inclusion criteria for the families included in this study.

Inclusion Criteria	Families *n*
Three or more family members with breast and/or ovarian cancer	121 (27.5%)
Two family members with breast and/or ovarian cancer	148 (33.6%)
Single affected individual with breast cancer ≤35 years of age	87 (19.8%)
Single affected individual with breast cancer between 36 and 50 years of age	84 (19.1%)
Total	440 (100%)

**Table 2:** Genotype and allelic frequencies of rs3746444, rs2910164, rs12975333, rs2043556, and rs4541843 in *BRCA1/2*-negative breast cancer cases and controls.

		All BC Cases (*n* = 440)			Families with ≥2 BC and/or OC Cases (*n* = 269)			Families with a Single Case, Diagnosis at ≤50 Years of Age (*n* = 171)		
Genotype or Allele	Controls (*n* = 1048) (%)	BC Cases (%)	*p-*Value ^a^	OR [95% CI]	BC Cases (%)	*p-*Value ^a^	OR [95% CI]	BC Cases (%)	*p-*Value ^a^	OR [95% CI]
rs3746444 (Pre-miR-499)										
A/A	772 (73.7)	319 (72.5)	-	1.0 (Ref)	198 (73.6)	-	1.0 (Ref)	121 (70.8)	-	1.0 (Ref)
A/G	254 (24.2)	111 (25.2)	0.6	1.0 [0.8–1.3]	64 (23.8)	0.9	0.9 [0.7–1.3]	47 (27.5)	0.3	1.1 [0.8–1.7]
G/G	22 (2.1)	10 (2.3)	0.8	1.1 [0.5–2.3]	7 (2.6)	0.6	1.2 [0.5–2.9]	3 (1.7)	1.0	0.8 [0.2–2.9]
A/G + G/G	276 (26.3)	121 (27.5)	0.6	1.0 [0.8–1.3]	71 (26.4)	1.0	1.0 [0.7–1.3]	50 (29.2)	0.4	1.1 [0.8–1.6]
Allele A	1798 (85.8)	749 (85.1)	-	1.0 (Ref)	460 (85.5)	-	1.0 (Ref)	289 (84.5)	-	1.0 (Ref)
Allele G	298 (14.2)	131 (14.9)	0.6	1.0 [0.8–1.3]	78 (14.5)	0.9	1.0 [0.7–1.3]	53 (15.5)	0.5	1.1 [0.8–1.5]
rs2910164 (Pre-miR-146a)										
G/G	561 (53.5)	236 (53.6)	-	1.0 (Ref)	149 (55.4)	-	1.0 (Ref)	87 (50.9)	-	1.0 (Ref)
G/C	410 (39.1)	165 (37.5)	0.7	0.9 [0.7–1.1]	101 (37.5)	0.6	0.9 [0.6–1.2]	64 (37.4)	1.0	1.0 [0.7–1.4]
C/C	77 (7.4)	39 (8.9)	0.3	1.2 [0.7–1.8]	19 (7.1)	0.8	0.9 [0.5–1.5]	20 (11.7)	0.06	1.6 [0.9–2.8]
G/C + C/C	487 (46.5)	204 (46.4)	1.0	0.9 [0.7–1.2]	120 (44.6)	0.6	0.9 [0.7–1.2]	84 (49.1)	0.5	1.1 [0.8–1.5]
Allele G	1532 (73.1)	637 (72.4)	-	1.0 (Ref)	399 (74.2)	-	1.0 (Ref)	238 (69.6)	-	1.0 (Ref)
Allele C	564 (26.9)	243 (27.6)	0.7	1.0 [0.8–1.2]	139 (25.8)	0.6	0.9 [0.7–1.1]	104 (30.4)	0.2	1.1 [0.9–1.5]
rs12975333 (Pre-miR-125a)										
G/G	1040 (99.2)	436 (99.1)	-	1.0 (ref)	267 (99.3)	-	1.0 (ref)	169 (98.8)	-	1.0 (ref)
G/T	8 (0.8)	4 (0.9)	0.7	1.1 [0.3–3.9]	2 (0.7)	0.2	1.9 [0.5–6.5]	2 (1.2)	0.6	1.5 [0.3–7.3]
T/T	0	0	-	-	0	-	-	0	-	-
G/T + T/T	8 (0.8)	4 (0.9)	0.7	1.1 [0.3–3.9]	2 (0.7)	0.2	1.9 [0.5–6.5]	2 (1.2)	0.6	1.5 [0.3–7.3]
Allele G	2088 (99.6)	876 (99.5)	-	1.0 (ref)	536 (99.6)	-	1.0 (ref)	340 (99.4)	-	1.0 (ref)
Allele T	8 (0.4)	4 (0.5)	0.7	1.1 [0.3–3.9]	2 (0.4)	0.2	1.9 [0.5–6.5]	2 (0.6)	0.6	1.5 [0.3–7.3]
rs2043556 (miR-605)										
T/T	376 (35.9)	208 (47.3)	-	1.0 (ref)	128 (47.6)	-	1.0 (ref)	80 (46.8)	-	1.0 (ref)
T/C	571 (54.5)	182 (41.3)	<10^−4^	0.5 [0.4–0.7]	115 (42.7)	0.0003	0.5 [0.4–0.7]	67 (39.2)	0.0009	0.5 [0.3–0.7]
C/C	101 (9.6)	50 (11.4)	0.6	0.8 [0.6–1.3]	26 (9.7)	0.2	0.7 [0.5 –1.2]	24 (14.0)	0.6	1.1 [0.6–1.8]
T/C + C/C	672 (64.1)	232 (52.7)	<10^−4^	0.6 [0.4–0.7]	141 (52.4)	0.0006	0.6 [0.4–0.8]	91 (53.2)	0.02	0.6 [0.5–0.9]
Allele T	1323 (63.1)	598 (68.0)	-	1.0 (ref)	371 (69.0)	-	1.0 (ref)	227 (66.4)	-	1.0 (ref)
Allele C	773 (36.9)	282 (32.0)	0.01	0.8 [0.6–0.9]	167 (31.0)	0.01	0.7 [0.6–0.9]	115 (33.6)	0.4	0.9 [0.7–1.1]
rs4541843 (Pri-miR-182)										
C/C	386 (36.8)	150 (34.1)	-	1.0 (Ref)	81 (30.1)	-	1.0 (ref)	69 (40.4)	-	1.0 Ref
C/T	473 (45.1)	205 (46.6)	0.4	1.1 [0.8–1.4]	127 (47.2)	0.1	1.2 [0.9–1.7]	78 (45.6)	0.6	0.9 [0.6–1.3]
T/T	189 (18.1)	85 (19.3)	0.4	1.5 [0.8–1.5]	61 (22.7)	0.03	1.5 [1.0–2.2]	24 (14.0)	0.1	0.7 [0.4–1.1]
C/T + T/T	662 (63.2)	290 (65.9)	0.3	1.1 [0.8–1.4]	188 (69.9)	0.04	1.3 [1.0–1.8]	102 (59.6)	0.3	0.8 [0.6–1.1]
Allele C	1245 (59.4)	505 (57.4)	-	1.0 (Ref)	289 (53.7)	-	1.0 (ref)	216 (63.2)	-	1.0 (Ref)
Allele T	851 (40.6)	375 (42.6)	0.3	1.0 [0.9–1.2]	249 (46.3)	0.01	1.2 [1.0–1.5]	126 (36.8)	0.2	0.8 [0.6–1.0]

BC—breast cancer; OC—ovarian cancer; OR—odds ratio; CI—confidence interval; Ref—Reference. ^a^ Fisher’s exact test. *p* < 0.05 statistically significant.

**Table 3:** Genotype and allelic frequencies of rs2043556 and rs4541843 according the number of BC cases in the families in *BRCA1/2*-negative breast cancer cases and controls.

		Families with 2 BC and/or OC Cases (*n* = 148)			Families with ≥3 BC and/or OC Cases (*n* = 121)		
Genotype or Allele	Controls (*n* = 1048) (%)	BC Cases (%)	*p*-Value ^a^	OR [95% CI]	BC Cases (%)	*p-*Value ^a^	OR [95% CI]
rs2043556 (miR-605)							
T/T	376 (35.9)	67 (45.3)	-	1.0 (Ref)	61 (50.4)	-	1.0 (Ref)
T/C	571 (54.5)	68 (45.9)	0.08	0.7 [0.5–1.0]	47 (38.9)	0.003	0.5 [0.4–0.8]
C/C	101 (9.6)	13 (8.8)	0.3	0.7 [0.4–1.4]	13 (10.7)	0.5	0.7 [0.4–1.5]
T/C + C/C	672 (64.1)	81 (54.7)	0.06	0.7 [0.5–1.0]	60 (49.6)	0.006	0.5 [0.4–0.9]
Allele T	1323 (63.1)	202 (68.2)	-	1.0 (Ref)	169 (69.8)	-	1.0 (Ref)
Allele C	773 (36.9)	94 (31.8)	0.1	0.8 [0.6–1.0]	73 (30.2)	0.06	0.7 [0.5–1.0]
rs4541843 (Pri-miR-182)							
C/C	386 (36.8)	45 (30.4)	-	1.0 (Ref)	36 (29.8)	-	1.0 (Ref)
C/T	473 (45.1)	70 (47.3)	0.2	1.2 [0.8–1.8]	57 (47.1)	0.2	1.2 [0.8–1.9]
T/T	189 (18.1)	33 (22.3)	0.1	1.4 [0.8–2.4]	28 (23.1)	0.09	1.5 [0.9–2.6]
C/T + T/T	662 (63.2)	103 (69.6)	0.1	1.3 [0.9–1.9]	85 (70.2)	0.1	1.3 [0.9–2.0]
Allele C	1245 (59.4)	160 (54.1)	-	1.0 (Ref)	129 (53.3)	-	1.0 (Ref)
Allele T	851 (40.6)	136 (45.9)	0.09	1.2 [0.9–1.5]	113 (46.7)	0.07	1.2 [0.9–1.6]

BC—breast cancer; OC—ovarian cancer; OR—odds ratio; CI—confidence interval; Ref—Reference. ^a^ Fisher’s exact test. *p* < 0.05 Statistically significant.

**Table 4:** Combined effects of rs3803662 (*TOX3*) and rs4541843 (pri-miR-182) on the risk of breast cancer.

Number of Risk Alleles ^(a)^	Controls (*n* = 1048) (%)	All BC Cases (*n* = 440)			Families with ≥2 BC and/or OC cases (*n* = 269)			Families with a Single Case, Diagnosis at ≤50 Years of Age (*n* = 171)		
		BC Cases (%)	OR [95% CI]	*p-*Value ^(b)^	BC Cases (%)	OR [95% CI]	*p-*Value ^(b)^	BC Cases (%)	OR [95% CI]	*p-*Value ^(b)^
0 risk alleles	153 (14.6)	49 (11.1)	1.0 (Ref)	-	22 (8.2)	1.0 (Ref)	-	27 (15.8)	1.0 (Ref)	-
1 risk allele	381 (36.4)	128 (29.1)	1.0 [0.7–1.5]	0.8	73 (27.1)	1.3 [0.7–2.2]	0.3	55 (32.2)	0.8 [0.4–1.3]	0.4
2 risk alleles	336 (32.1)	168 (38.2)	1.5 [1.0–2.2]	0.01	105 (39)	2.1 [1.3–3.5]	0.001	63 (36.9)	1.0 [0.6–1.7]	0.9
3 risk alleles	153 (14.6)	79 (18)	1.6 [1.0–2.4]	0.02	57 (21.2)	2.5 [1.5–4.4]	0.0006	22 (12.9)	0.8 [0.4–1.4]	0.5
4 risk alleles	25 (2.4)	16 (36)	1.9 [0.9–3.8]	0.08	12 (4.5)	3.2 [1.4–7.2]	0.006	4 (2.3)	0.8 [0.2–2.6]	1.0
*p*-trend ^(c)^				0.0005			<10^−4^			0.9755
Global *p* ^(d)^				0.005			0.0001			0.6970

^(a)^ 0 risk allele: C/C + C/C; 1 risk allele: C/C + C/T, C/T + C/C; 2 risk alleles: C/C + T/T, T/T + C/C, C/T + C/T; 3 risk alleles: C/T + T/T, T/T + C/T; 4 risk alleles: T/T + T/T. ^(b)^ Fisher’s exact test. ^(c)^ Chi-test for trend. ^(d)^ Chi-squared test for independence. BC—breast cancer; OC—ovarian cancer; OR—odds ratios; CI—confidence interval; Ref—Reference. *p* ≤ 0.05 statistically significant.

**Table 5:** Combined effects of rs38033662 (*TOX3*) and rs4541843 (pri-miR-182) on the risk of breast cancer according the number of BC cases in the families.

Number of Risk Alleles ^(a)^	Controls (*n* = 1048) (%)	Families with Two BC and/or OC Cases (*n* = 148)			Families with ≥3 BC and/or OC Cases (*n* = 121)		
		BC Cases (%)	OR [95% CI]	*p-*Value ^(b)^	BC Cases (%)	OR [95% CI]	*p-*Value ^(b)^
0 risk alleles	153 (14.6)	10 (6.8)	1.0 (Ref)	-	12 (8.1)	1.0 (Ref)	-
1 risk allele	381 (36.4)	29 (19.6)	1.1 [0.5–2.4]	0.8	44 (29.7)	1.4 [0.7–2.8]	0.2
2 risk alleles	336 (32.1)	47 (31.8)	2.1 [1.0–4.3]	0.03	58 (39.2)	2.2 [1.1–4.2]	0.01
3 risk alleles	153 (14.6)	30 (20.3)	3.0 [1.4–6.3]	0.003	27 (18.2)	2.2 [1.0–4.6]	0.02
4 risk alleles	25 (2.4)	5 (3.4)	2.9 [0.9–9.3]	0.06	7 (4.7)	3.4 [1.2–9.5]	0.02
*p*-trend^(c)^				0.0001			0.001
Global *p* ^(d)^				0.001			0.02

^(a)^ 0 risk allele: C/C + C/C; 1 risk allele: C/C + C/T, C/T + C/C; 2 risk alleles: C/C + T/T, T/T + C/C, C/T + C/T; 3 risk alleles: C/T + T/T, T/T + C/T; 4 risk alleles: T/T + T/T. ^(b)^ Fisher’s exact test. ^(c)^ Chi-test for trend. ^(d)^ Chi-squared test for independence. BC—breast cancer; OC—ovarian cancer; OR—odds ratios, CI—confidence interval; Ref—Reference. *p* ≤ 0.05 Statistically significant.
